# Prediction and Regulation of SCC’s Shrinkage Using the PSO-BPNN Model

**DOI:** 10.3390/ma19071468

**Published:** 2026-04-07

**Authors:** Tongyuan Ni, Lihua Shen, Shenghao Shen, Zaoyang Cai, Wen Chu, Chengshun Hu, Chenhui Jiang, Kai Jing

**Affiliations:** 1 College of Civil Engineering, Zhejiang University of Technology, Hangzhou 310023, China; 221123060116@zjut.edu.cn (L.S.); 221124060115@zjut.edu.cn (S.S.); 221125060156@zjut.edu.cn (Z.C.); 221122060117@zjut.edu.cn (W.C.); 2 Zhejiang Key Laboratory of Green Construction and Intelligent Operation & Maintenance for Coastal Infrastructure, Hangzhou 310023, China; 3 Zhejiang Infrastructure Construction Group Co., Ltd., Hangzhou 310012, China; 4 Department of Construction Engineering, Zhejiang College of Construction, Hangzhou 311231, China; chjiang2020@126.com; 5 Zhejiang Provincial Yijian Construction Group Co. Ltd., Hangzhou 310013, China

**Keywords:** self-compacting concrete (SCC), concrete-filled steel tube (CFST), autogenous shrinkage, regulation, Particle Swarm Optimization-Back Propagation Neural Networks (PSO-BPNN)

## Abstract

The shrinkage deformation is a significant risk to self-compacting concrete (SCC)-filled steel tube structures. It was essential to understand the concrete autogenous shrinkage strain before being regulated in order to determine compensation shrinkage measures. In this study, A PSO-BPNN model was constructed, which is based on the Particle Swarm Optimization-Back Propagation Neural Networks (PSO-BPNN), and the autogenous shrinkage strain of SCC was predicted based on PSO-BPNN before being regulated. Moreover, some experiments about compensating for shrinkage by expansion and by a combination of expansion and contraction were investigated. Based on this prediction, a series of experiments was conducted on the regulation of the shrinkage deformation of SCC for an actual bridge project. The results indicated that a good consistency of PSO-BPNN between predicted and measured values, demonstrating that PSO-BPNN is a model with high accuracy in predicting concrete autogenous shrinkage strain before regulation, and as a guidance for regulation to compensate for shrinkage. The prediction error was less than 10% for 28-day self-shrinkage, and the experimental workload was reduced. The PSO-BPNN is a convenient tool for predicting the shrinkage of SCC, enabling the determination of dosages of expansion agent and reducing shrinkage agent to achieve SCC’s shrinkage regulation.

## 1. Introduction

Self-compacting concrete (SCC)-filled steel tube structure is a type of composite structure that is composed of two basic parts, i.e., the hollow tube/pipe made with carbon steel and the infilled self-compacting concrete. The peripheral steel tube/pipe confines the core concrete, causing a cyclo-hoop effect on the concrete, which enhances the structure’s overall stiffness and load-carrying capacity [1,2,3]. The SCC-filled steel tube structures are currently widely utilized in high-rise buildings, large-scale structures, subway stations, and even military engineering. The structural performance, especially the synergy of steel tube/pipe and core of SCC, is undermined by the volume changes in SCC [4,5]. The volume changes in SCC mainly result from hydration-induced autogenous shrinkage and thermal strain, along with drying shrinkage caused by evaporation of free water in capillary pores [6,7,8]. It should be noted that, since SCC is entirely enclosed by the steel tube, the drying shrinkage can be substantially ignored [9,10,11,12,13]. The net result of volume changes in SCC is shrinkage. This kind of shrinkage is detrimental to SCC elements and structures, such as the core concrete, which is peeled from the pipe/tube inner wall. It inevitably causes the cyclo-hoop effect to not work. In turn, the bearing capacity of the structures cannot meet the service requirements, and further becomes a potential hazard [14].

Some common ways were used to solve it in engineering practice, such as booster pump concrete, open holes to fill the gap between the pipe/tube and concrete, etc. [10,15] Unluckily, these methods all have their own shortcomings. To boost pump concrete, small pressure does not solve the problem, and higher pressure may lead to injury to the steel pipe/tube or construction difficulties [4,8,16,17]. Obviously, it is bound to hurt the steel pipe/tube to open holes to fill the gap between the pipe/tube and the concrete. The engineer does not recommend such a remedial technique.

**Figure 1:**
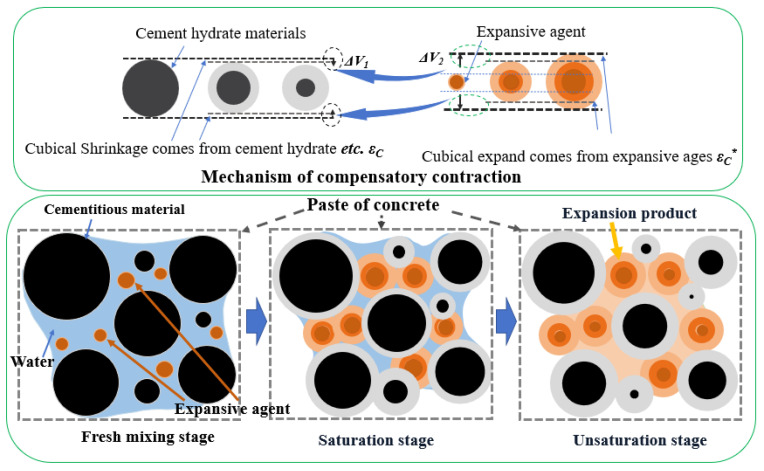
Mechanism schematic diagram to compensate for concrete shrinkage. Note: ε*—strain after compensation.

Another way to reduce the shrinkage of SCC is to add special admixtures, such as shrinkage-reducing agents and expansion agents [15,18,19,20,21]. The shrinkage-reducing admixture (SRA) successfully mitigates the shrinkage in concrete by reducing the surface tension of the liquid phase in the capillary pores [22]. Rong-bing and Jian [23] found that the addition of 3% SRA reduced the drying shrinkage of the concrete by 44% at 90 days. Zuo et al. [24] added 2% SRA, which is composed of low molecular polyether, to a cement paste to reduce autogenous shrinkage to 55% after 7 days. These beneficial effects of SRA can be attributed to the alteration of the cement microstructure, the reduction in hydration rate, and the surface tension of the pore solution. However, the autogenous shrinkage cannot be eliminated completely. Shrinkage-compensating admixture (SCA), such as expansion agent (EA) in powder form, is also frequently used in self-compacting mixtures containing a high content of cement and a lower water-cement ratio [25]. In theory, the shrinkage can be fully compensated, even though concrete shows slightly expanded, i.e., *V*_2_ can compensate for ∆*V*_1_ completely, as shown in Figure 1. And the volume expansion *V*_2_ comes from the expansive agent hydrate product’s volume increasing. However, the dosage of admixtures such as SCA and EA is typically determined through experimentation, which requires a substantial amount of experimental work. In recent years, Artificial Neural Network (ANN) technology has been utilized to assist in determining the admixture dosage [26,27], and the ANN algorithm is also undergoing continuous improvement and evolution.

It is highly challenging work to accurately predict concrete shrinkage. There are two typical kinds of prediction models for concrete self-shrinkage: one was established through a causal relationship between the objective phenomena of concrete materials and the mechanisms, such as Tazawa [28], and the other was established through fundamental properties. The other develops empirical formulas based on statistical analysis of extensive experimental data, such as the AS3600-2018 model [29], the improved B4 model [30], and the elastic hypothesis model [31]. Based on demonstrated broad applicability due to their high flexibility and adaptability in input and output parameters of the Artificial Neural Networks (ANNs), this may enable the aforementioned two types of models to achieve integration. Research on concrete performance prediction using ANN technology has demonstrated favorable results and significantly reduced experimental workload, such as concrete creep [32], shrinkage [33], and tensile performance [34]. In this study, A PSO-BPNN model was constructed, which is based on the Particle Swarm Optimization-Back Propagation Neural Networks (PSO-BPNN), and the concrete autogenous shrinkage strain before regulation was predicted based on PSO-BPNN. Moreover, some experiments about compensating for shrinkage by expansion and by a combination of expansion and contraction were investigated.

## 2. Prediction of Shrinkage Deformation Value of SCC Based on PSO-BPNN

It is a very difficult task to predict the shrinkage deformation value of SCC accurately. Some mechanistic concrete shrinkage models based on thermodynamic approach theory or micro-mechanical physics needed a large amount of test data [35,36,37,38,39,40], and were significantly influenced by external factors such as differences in materials and testing methods. The mechanism and influencing factors of concrete shrinkage are very complex. One kind of model cannot exhibit good generalization due to conditional constraints. It is a new attempt to explore a novel prediction method based on different experimental results using artificial neural network technology.

**Figure 2:**
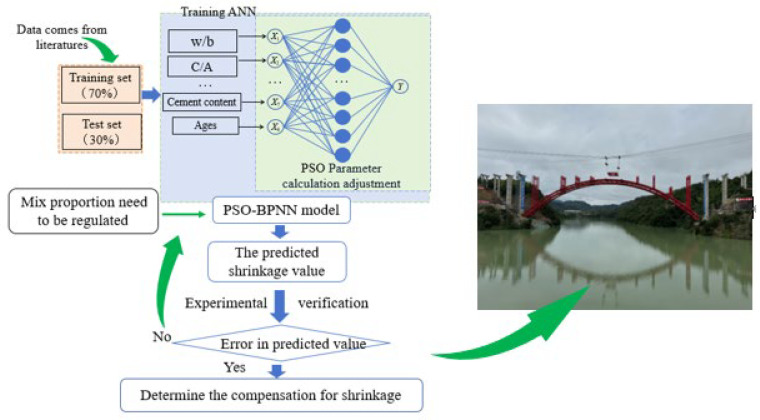
Prediction, regulation, and application flow chart of SCC shrinkage.

A basic database was established in this study using 4165 sets of experimental data from the team’s research tests during recent years [39] and the literature data collected by the research team [34] (Supplementary Material, Table S1: Database). The categories data sets were divided into a training set (30%) and a test set (70%), respectively; the training set and test set are randomly determined. After normalization, ten factors—cement content, water-to-binder ratio, cement-to-aggregate ratio, fly ash content, silica fume content, GGBFS content, curing temperature, curing humidity, and age—were selected as input parameters, and autogenous shrinkage of the concrete was set as the output parameter. A PSO-BPNN model was constructed, which is based on the Particle Swarm Optimization-Back Propagation (PSO-BP) [37], and the pre-regulation of the SCC shrinkage in the Hongkou Bridge project was predicted using this model for the purpose of providing a reference and scientific basis for further research. That is, the shrinkage behavior of concrete without being regulated will be predicted using the PSO-BPNN model, which is based on autogenous shrinkage data of concrete with various mix proportions collected from the literature [34], and then the dosage of expansive agents to compensate for expansion and regulate concrete performance was developed. Figure 2 shows the prediction, regulation, and application flow chart of SCC shrinkage.

### 2.1. PSO-BPNN Model

#### 2.1.1. The Parameters of the PSO-BPNN Setting

The first step was to confirm the input variables, which include cement dosage, water/cement ratio, aggregate/cement ratio, fly ash dosage, silica ash dosage, mineral powder dosage, volume to surface ratio, curing temperature, curing humidity, and age. Accordingly, the number of neurons in the input layer of PSO-BPNN was set to 10.

The learning rate is the hyperparameter of the control re-update step, which determines the update amplitude of the model in each iteration. A large learning rate can cause the model to converge quickly, but also lead to shock or skipping the optimal solution; a smaller learning rate can make the model converge more stably, but may lead to long training time. In this paper, the initial learning rate was set to 0.01 to avoid prematurely stopping training or over-training.

By monitoring the loss function on the validation set, terminate the training prematurely when the validation loss no longer drops, thus avoiding over-fitting. This paper sets the maximum number of training times to 1000 times.

The implied level node is set according to the following empirical formula, i.e., Equation (1).

(1) $$j=\sqrt{a+p}+c$$

where *j* represents the number of neurons in the hidden layer of the neural network, *a* represents the number of neurons in the input layer, *p* represents the number of neurons in the output layer, and *c* is an integer between 1 and 10. The root-mean-square error (RMSE), analyzing the errors between the predicted and experimental values, was calculated according to Equation (2).

(2) $$\mathrm{RMSE}=\sqrt{\frac{1}{M_{a}}\sum_{i=1}^{M_{a}}{\left(\frac{A_{a}-A_{0}}{A_{a}}\right)}^{2}}$$

where *M_a_* is the number of experimental data samples, *A_a_* is the actual value (target value), and *A*_0_ is the predicted value of the model.

**Figure 3:**
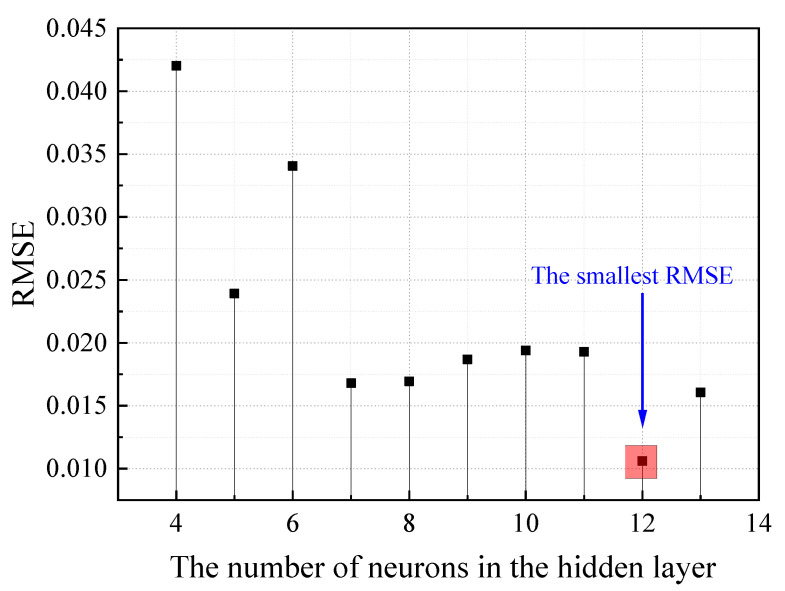
The RMSE of hidden layer neurons with different numbers.

The number of hidden layer neurons can be selected when the smallest RMSE, because the neural network performance was the best. The RMSE of hidden layer neurons with different numbers in this study can be shown in Figure 3.

#### 2.1.2. Optimized Parameter Setting

The initial weights and thresholds of the BPNN were optimized by the PSO algorithm to improve the prediction accuracy of the model. The main parameters of the PSO algorithm include population size *M_b_*, number of iterations *N*, particle maximum velocity *V_max_*, inertial weight *w_a_*, and acceleration constants *c*_1_ and *c*_2_. Consider the local search ability, global search ability of particles (parameters: *V_max_*, *w_a_*), and the self-learning ability of PSO-BPNN (*c*_1_, *c*_2_). The parameters in this study were determined as *M_b_* = 20, *N* = 50, *V_max_* = 3, *w_a_* ∈ [0.4, 0.9], *c*_1_ = *c*_2_ = 2.

### 2.2. Prediction of Shrinkage of SCC by the PSO-BPNN Model and Experimental Validation

#### 2.2.1. Prediction

The mixed proportion of control concrete is listed in Table 1.

**Figure 4:**
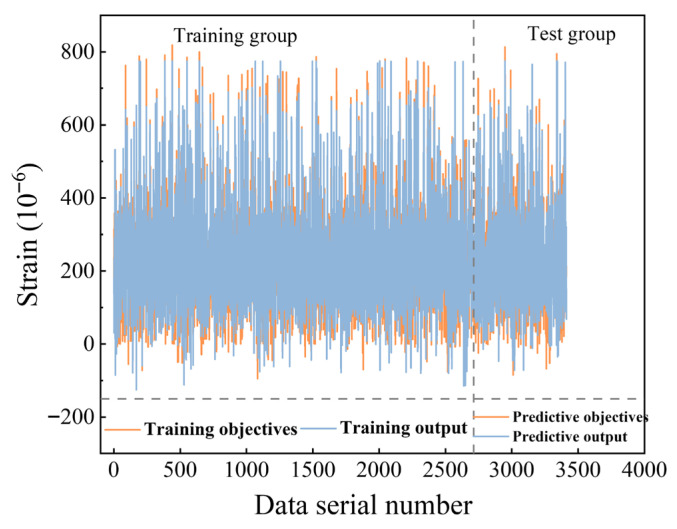
Results of comparison of model predictions with true values.

**Figure 5:**
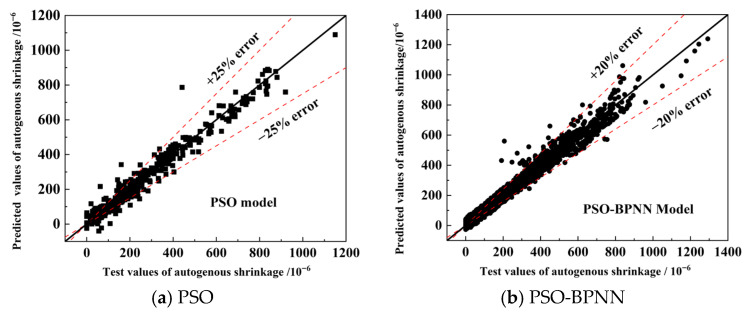
Results of comparison between experimental and predicted values of the PSO and PSO-BPNN model.

In order to know the concrete autogenous shrinkage strain before regulation, the concrete autogenous shrinkage was predicted based on the PSO-BPNN model, and it reaches the error target limit after 10 times PSO-BPNN training. The results of the test values compared with the predicted output values of PSO-BPNN are shown in Figure 4. The results of the comparison between experimental and predicted values of the PSO and PSO-BPNN models are shown in Figure 5, and the error of predicted values of the PSO-BPNN model was reduced 5% (from 25% to 20%). The prediction accuracy of the improved model PSO-BPNN was significantly improved.

#### 2.2.2. Experimental Validation of PSO, PSO-BPNN Prediction

**Figure 6:**
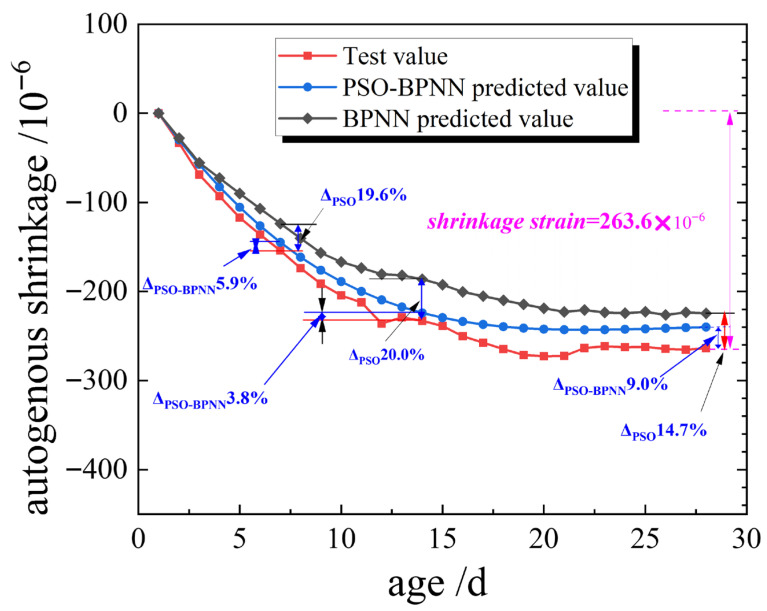
Comparison with the predicted and measured values of autogenous shrinkage.

In order to validate the prediction accuracy of PSO and PSO-BPNN on autogenous shrinkage of unregulated concrete, and obtain the autogenous shrinkage value, the concrete autogenous shrinkage experiments were investigated. The mixed proportion of the unregulated concrete is shown in Table 1, and the results of that are shown in Figure 6. The predicted absolute value of autogenous shrinkage at the age of 28 days based on PSO-BPNN for concrete with unregulated mix proportions was −239.9 × 10^−6^, while the experimental measurement showed an absolute value of −263.6 × 10^−6^. Throughout the 28-day period, the difference between predicted and measured values remained within 30 × 10^−6^. The percentage differences in test values between PSO-BPNN at 7, 14, and 28 days were 5.9%, 3.8%, 9.0%, all below 10%. And the percentage differences in test values between PSO were 19.6%, 20.0%, and 14.7%, respectively. These indicate good consistency of PSO-BPNN between predicted and measured values, demonstrating the PSO-BPNN model’s high accuracy in predicting autogenous shrinkage. The errors primarily originate from two aspects: firstly, validation experiments-induced errors, such as variations in raw materials and experimental procedures; secondly, the other origin was from the model prediction errors. To validate the engineering applicability of PSO-BPNN, the predicted values (−263.6 × 10^−6^) were used as the quantitative measure for SCC compensation shrinkage.

## 3. Regulation of the Shrinkage Deformation of SCC

### 3.1. Proportion Design and Regulation

The successful preparation of micro-expansion SCC should meet four basic conditions [41,42,43,44,45]:

(i). To produce sufficient expansion compensation for shrinkage during the early age;
(ii). To ensure the harmlessness of SCC during the delayed expansion;
(iii). To compensate for the long-term and sustainable shrinkage of SCC after the expansion reaction;
(iv). To reasonably select the type and dosage of shrinkage-compensating materials, so as to avoid the excessive negative impact on SCC caused by delayed expansion or harmful effects.

So, in order to reduce the risk of interface debonding resulting from the volume deformation of SCC, such as autogenous shrinkage, and optimize the volume stability of SCC, the mix proportion of concrete was listed in Table 2. Based on the expansion agent (EA) performance and the shrinkage strain, for which PSO-BPNN prediction was −263.6 × 10^−6^, the dosages of expansion agent were 8%, 9%, and 10% by equal mass replacement cement. The shrinkage reducing agent (SRA) was mixed by the external mixing method, and the water glue ratio, aggregate content, and sand rate remained unchanged as an unregulated reference group. In this paper, the water/binding ratio is 0.32, and the sand ratio is 0.41.

### 3.2. Shrinkage Compensation by Expansion Agents

**Figure 7:**
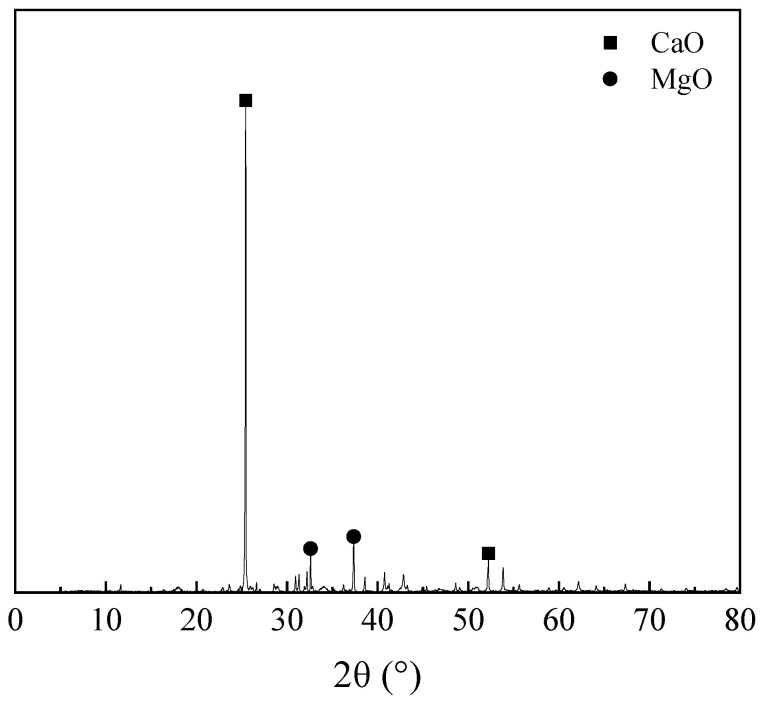
XRD pattern of EA.

The influence of EA is to determine the reasonable dosage of EA in SCC, and how the influence of different dosage of EA on SCC working performance, mechanical properties and volume deformation performance were investigated, in order to explore the reasonable dosage combining with the preliminary prediction results, and moreover to determine the micro-expansion SCC mix proportion to ensure the stability and reliability of concrete in the service process. The XRD pattern of EA is shown in Figure 7.

The experiment employed prismatic specimens measuring 100 mm × 100 mm × 400 mm with two parallel specimens per group. All specimens were placed inside the laboratory room. The experiment was conducted in the environment at a temperature of (20 ± 2) °C and a humidity of (60 ± 5) % RH. The time of concrete pouring into the mold is taken as the zero point for shrinkage testing.

**Figure 8:**
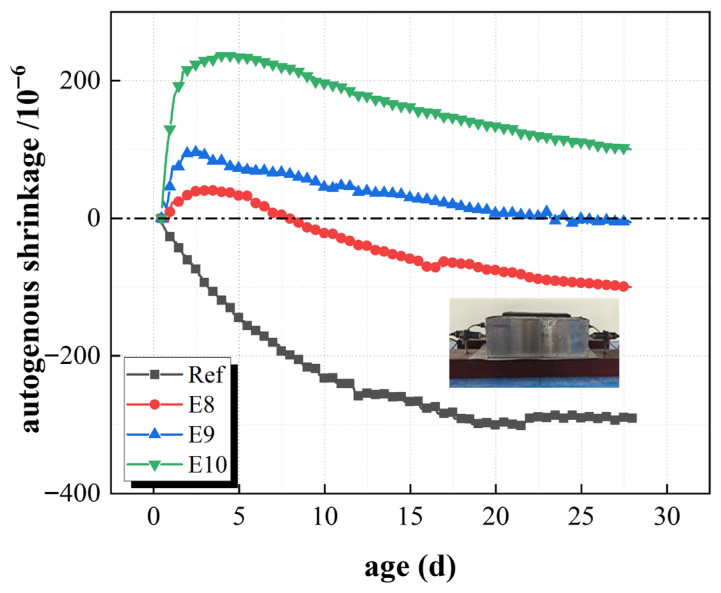
Autogenous shrinkage of unconstrained concrete with admixture EA.

Figure 8 shows the shrinkage deformation of SCC mixed with EA and without EA. The control group contracted continuously after the final setting, and the shrinkage was −290.5 × 10^−6^ at the age of 28 d. However, the value of experimental validation was −263.6 × 10^−6^, the difference is clearly caused by asynchronous experiments, and it also indicates that parallel specimens are necessary. The concrete mixed with EA (group E_8_, E_9_, E_10_) showed significant expansion after the final setting. The expansion stage ended after about 3.5 days, and the expansion strain amounts were 40.5 × 10^−6^, 97.2 × 10^−6,^ and 229.1 × 10^−6^, respectively. The reason for expansion was that the incorporation of EA produced more AFt and Ca(OH)_2_ crystals, and these expansion products would compensate for the shrinkage strain caused by the hydration of cementitious material at early ages, and even produce expansion [46,47,48,49]. The results also showed that the expansion of the E_10_ group is significantly higher than that of the E_8_ group, while the amount of EA incorporation increases. The concrete showed continuous contraction, but the contraction rate was slower than that of the Ref. group because of expansion at this stage. The strain development of concrete autogenous shrinkage tended to be stable after 28 d, and the autogenous deformation strain values of Ref. group, E_8_, E_9_, and E_10_ were −290.5 × 10^−6^, −100.0 × 10^−6^, −5.6 × 10^−6^, and 99.9 × 10^−6^, respectively. The concrete autogenous shrinkage of the E_8_ and E_9_ groups at 28 d was decreased 65% and 98%, respectively, while the E_10_ group achieved a micro expansion to 99.9 × 10^−6^ at the same age. These results indicated that the incorporation of a higher content of EA can effectively compensate for autogenous shrinkage and achieve expansion compensation. Therefore, the reasonable selection of the expansion agent can help to improve the volume stability of concrete, slow down, and overcome the problem of shrinkage.

**Figure 9:**
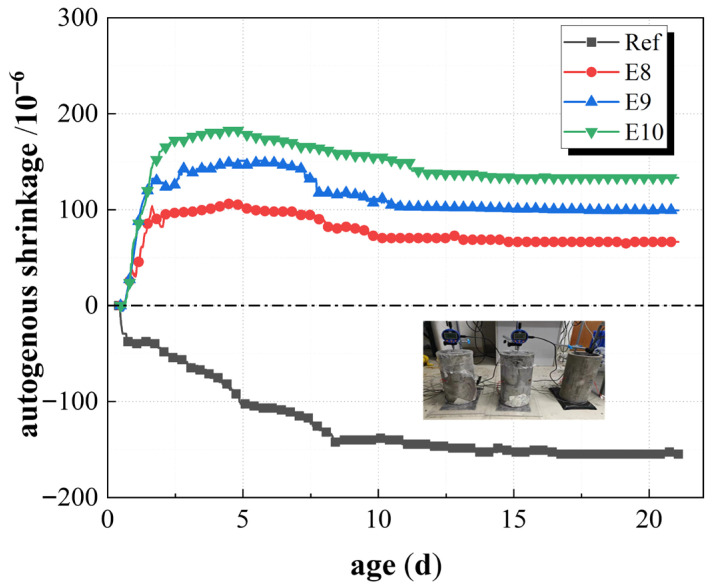
Autogenous shrinkage of concrete with EA restrained by a steel tube.

The autogenous shrinkage of concrete with EA, which was constrained by steel pipes, is shown in Figure 9. The Ref. group concrete continued to shrink after the initial condensation, and the shrinkage deformation tended to stabilize after about 14 days. The shrinkage deformation strain at the age of 21 d was −154.8 × 10^−6^, which decreased by 48.4% since the autogenous shrinkage compared to the unconstrained state. The significant expansion was shown in the E_8_, E_9_, and E_10_ groups. After initial condensation, the expansion stage ended after 3 days, and the expansion strain values of the three groups were 99.8 × 10^−6^, 142.6 × 10^−6^, and 180.4 × 10^−6^, respectively. After about 3 days, the expansion phase of concrete continued to show a continuous contraction.

**Figure 10:**
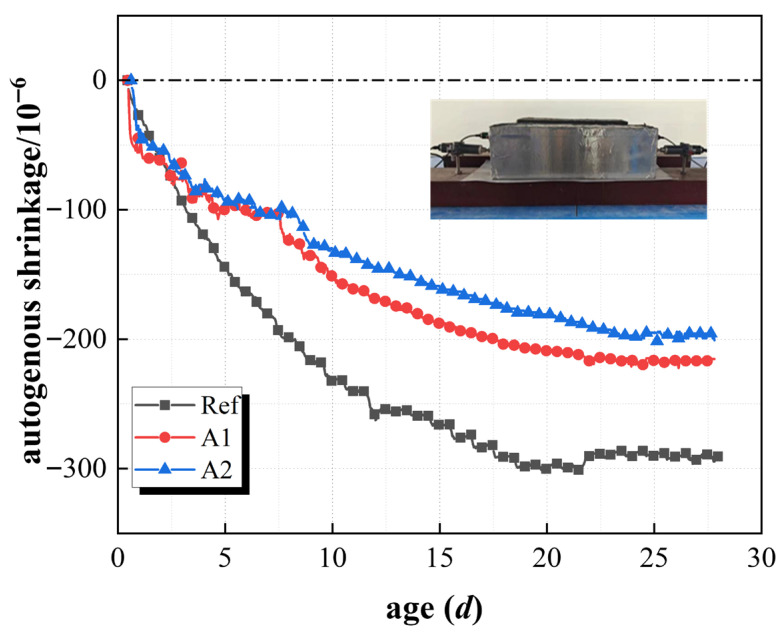
Autogenous shrinkage of unrestrained concrete with SRA.

Figure 10 shows the strain caused by concrete volume deformation with SRA in the unconstrained state. The results showed that the autogenous shrinkage strain value of the Ref. group at the age of 28 d was −290.5 × 10^−6^. In contrast, the autogenous shrinkage strain values of groups A_1_ and A_2_ were −215.3 × 10^−6^ and −196.5 × 10^−6^, respectively. These results showed that the autogenous shrinkage strain of concrete could be reduced by the incorporation of SRA effectively, but not all of the shrinkage strain. Specifically, the shrinkage strain of the A_1_ group was 75.2 × 10^−6^ less than that of the Ref. group, and with a reduction of about 25.9%. The autogenous shrinkage strain value of the A_2_ group decreased by 94.0 × 10^−6^ from that of the Ref. group, with a reduction of about 32.3%. These results also showed that the autogenous shrinkage of the concrete with the incorporation of SRA significantly slowed down, but the reduction effect was more significant when the SRA incorporation was higher. The reason for these results was that the SRA would reduce the friction between cement particles in the surface activity and dispersion of concrete, and delay the speed of the cement hydration reaction. So, the reduction of autogenous shrinkage strain caused by the process of cement hydration [44,46]. The results of Figure 8 also showed that the autogenous shrinkage strain value of the A_2_ group (−196.5 × 10^−6^) was lower than that of group A_1_ (−215.3 × 10^−6^). This indicated that the higher SRA (the A_2_ group) was more effective. The autogenous shrinkage strain value of group A_2_ at 28 days decreased by 32.3% compared to the Ref. group, indicating that the reduction effect of shrinkage was more significant when the SRA incorporation was higher, and this effect would be more pronounced in the long term. In conclusion, the incorporation of SRA can effectively slow down the shrinkage of concrete, and the reduction effect of SRA was enhanced with the increase in incorporation. So, the reasonable selection of SRA can optimize the volume stability of concrete while ensuring its mechanical properties and improve its long-term crack resistance and durability.

### 3.3. Compensate for Shrinkage Through a Combination of Expansion and Contraction

**Figure 11:**
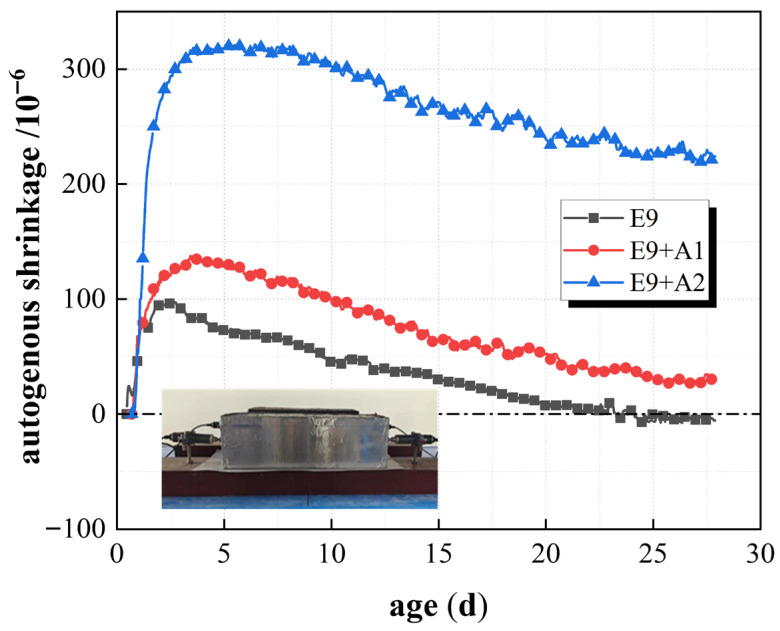
Autogenous shrinkage of unconstrained concrete with EA and SRA.

Figure 11 shows the shrinkage strain of unconstrained concrete, which is double incorporated with EA and SRA. The results showed that the shrinkage strain value of the E_9_ group was −5.6 × 10^−6^, whereas the E_9+A1_ and E_9+A2_ groups were 30.7 × 10^−6^ and 224.1 × 10^−6^, respectively, and it indicated that a significant change occurred in the volume deformation of the concrete after SRA and EA incorporation. Specifically, the autogenous shrinkage strain value of the E_9_ group was close to zero. It indicates that the shrinkage phenomenon almost does not happen, and that EA compensates for the whole shrinkage of concrete. However, the E_9+A1_ and E_9+A2_ groups with SRA and EA double incorporation showed a relatively significant expansion effect, especially the autogenous shrinkage strain value of the E_9+A2_ group at 28 days, which reached 224.1 × 10^−6^, indicating a significant expansion phenomenon. The SRA incorporation partly promotes volume expansion, especially at higher amounts (2%). The autogenous shrinkage value of the E_9+A1_ group was 30.7 × 10^−6^. Although the shrinkage strain was increased compared to the E_9_ group, the expansion was smaller than that of the E_9+A2_ group, and the effect of SRA incorporation on volume shrinkage deformation was correlated. The admixture of SRA can improve the working of concrete and reduce the hydration rate of cement particles [34]. In conclusion, the concrete containing EA and SRA showed different volume shrinkage deformation trends. The admixture of EA effectively compensates for concrete shrinkage, especially the shrinkage strain of the E_9_ group, which was almost zero, while the addition of SRA promotes concrete expansion to some extent, especially at higher levels (E_9+A2_ group). This phenomenon suggests that the simultaneous incorporation of EA and SRA may lead to concrete volume expansion, especially the incorporation at higher levels. Therefore, the reasonable regulation of the amount of expansion agent and reducing shrinkage agent is the key to ensuring the stability of concrete volume, especially in structures that need to control the autogenous shrinkage.

**Figure 12:**
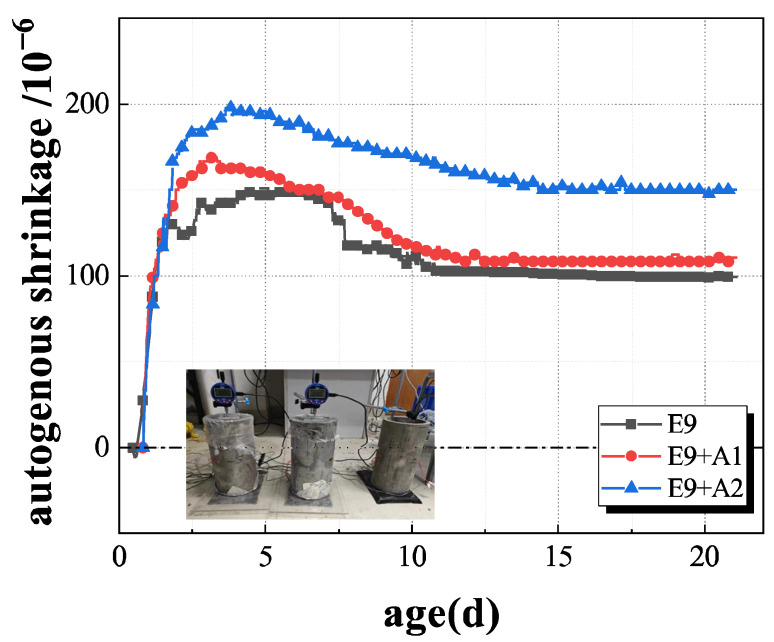
Autogenous shrinkage of concrete with EA and SRA constrained by a steel tube.

The volume deformation of concrete containing EA and SRA is shown in Figure 12. The autogenous shrinkage value of the E_9_ group at 28 d was 99.3 × 10^−6^, and the E_9+A1_ group and E_9+A2_ group were 110.4 × 10^−6^ and 150.0 × 10^−6^, respectively. Specifically, the volume deformation strain of the E_9+A1_ group expanded, up to 11.1 × 10^−6^ compared with the E_9_ group, an increase of about 11.2%. This indicates that the incorporation of SRA suppresses the autogenous shrinkage development of concrete, but the effect is moderate. The incorporation of 1% SRA caused a slight increase in volume shrinkage deformation of concrete, but did not show a significant expansion effect.

However, the volume deformation strain of the E_9+A2_ group reached 150.0 × 10^−6^ at 28 d of age, up 50.7 × 10^−6^ (about 51.1%) from that of the E_9_ group. This phenomenon shows that the concrete exhibits significant expansion effects at 2% SRA incorporation. From these results, it can be seen that the autogenous shrinkage of steel tube constrained concrete with double incorporation of EA and SRA was significantly reduced; that is, the incorporation of EA and SRA can not only promote the cement hydration reaction, but also reduce the shrinkage caused by hydration. While the EA plays the role of compensating for autogenous shrinkage, 2% of SRA can also reduce the shrinkage deformation of concrete, and the synergy between EA and SRA in concrete volume shrinkage deformation was good. In conclusion, the composite incorporation with EA and SRA can significantly change the volume deformation of steel tube constrained concrete, and the amount of SRA has a significant impact on the volume stability of concrete. Reasonable control of the mixing amount of SRA can effectively balance the autogenous shrinkage and expansion effect of concrete and improve the volume stability and long-term performance of concrete.

## 4. Results and Discussion

### 4.1. Guidance for Regulation Experiments Based on the Prediction of the PSO-BPNN Model

The optimized PSO-BPNN model is able to effectively capture the complex nonlinear relationship of autogenous shrinkage. Training and test results showed that the overall sample R^2^ reached 0.9543 after the training of the autogenous shrinkage prediction model. It shows that the training situation is good and the proposed model has good prediction performance.

The actual engineering concrete mix ratio data were input into the autogenous shrinkage prediction model, and the autogenous shrinkage value was obtained for 28 days after the prediction. After comparing the measured value of the concrete, the difference between the predicted value and the measured value of 7 d, 14 d, and 28 d was less than 10%, and this indicates that the predicted value and the measured value are consistent. The generalization ability and accuracy of concrete autogenous shrinkage were good.

According to the prediction model, the absolute value of the shrinkage of the concrete at 28 d age of the pre-regulation mix ratio is 239.9 × 10^−6^. In order to meet the requirement of SCC according to Technical Specification for Application of Self-compacting Concrete (T/CECS203-2021) [50], the volume expansion deformation of 340~440 × 10^−6^ should be provided in the subsequent regulation experiment, and the final self-contraction of the concrete should be controlled in the micro-expansion state of 100 × 10^−6^~200 × 10^−6^.

### 4.2. Effect of EA on Hydration Products of Concrete

In order to analyze the effect of expansive agent on hydration products of concrete, the content changes in Ca(OH)_2_ and chemically bound water in the paste sample of concrete at different ages were investigated by Thermogravimetric (TG) Analysis. The cement hydration products have their own decomposition temperature interval, and various DTG phases can be qualitatively identified by the TG-DTG curves, as shown by the DTG curves of the test samples at different ages. The TGA curve of typical cement hydration samples can be divided into three stages [51,52]: (1) water loss decomposition process of C-S-H gel, 300 °C; (2) 400–500 °C, mainly Ca(OH)_2_; and (3) decomposition of carbonate above 550 °C. In the DTG curve of the control group, the first weight loss peak occurs around 100 °C, mainly resulting from the water loss decomposition of C-S-H gel and calcium; the subsequent peak at 140 °C corresponds to the decomposition of gypsum in the cement clinker, which has completely disappeared after 6 h of hydration; the mono sulfur aluminate phase removes the interlayer water in the structure; the peak occurs 72 h after hydration; the peak at 400 °C to 500 °C is mainly due to the water loss decomposition of Ca(OH)_2_.

**Figure 13:**
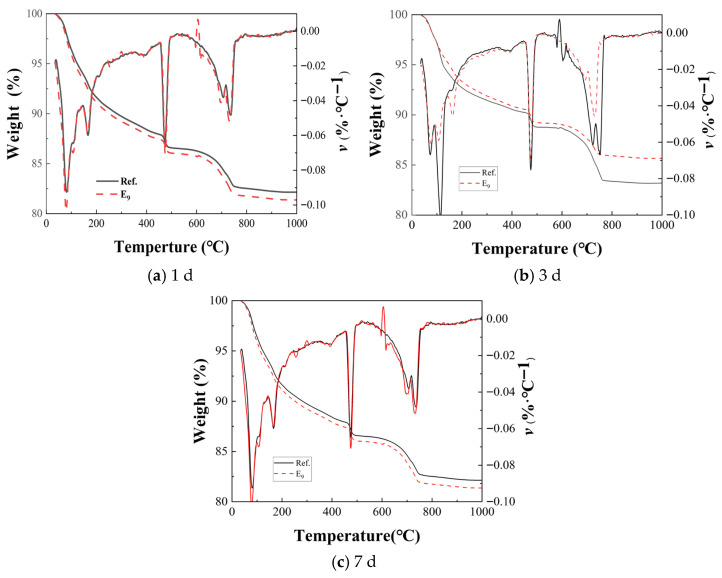
Thermogravimetry analysis of paste containing EA at different ages.

The thermogravimetry analysis results of the paste containing EA at different ages are shown in Figure 13. It can be found that the influence of EA on the volume deformation of the concrete hydration process was mainly at the start of day 7. It was reasonable that the TG analysis was performed in the 1 d, 3 d, and 7 d old groups to analyze the effect of EA on hydration products in concrete.

The TGA and DTG curves for the 1 d old of the Ref. E_9_ groups are shown in Figure 13a. The decrease in the paste was mainly caused by the evaporation of free water and the dehydration of the hydration products (mainly C-S-H). The mass of the paste drops sharply at temperatures of about 450 °C and 700 °C, which is caused by water evaporation that comes from the decomposition of Ca(OH)_2_ and CaCO_3_ decomposition of CO_2_ [44]. The Ref. and E_9_ groups showed a clear loss peak at 100 °C, which corresponds to the water loss decomposition of C-S-H gel and AFt (calcium). The weight loss peak seen at 140 °C corresponds to the decomposition of calcium sulfate, while the peak of the paste in the E_9_ group was significantly higher than the Ref. group, because the incorporation of EA replacing part of the cement could promote the generation of C-S-H gels, AFt, and CaSO4. The peak of Ca(OH)_2_ after EA alone was similar to that of the Ref. group; the reason for this was that the cement hydration speed of the E_9_ group was similar to that of the Ref. group at the age of 1 d, and the content of Ca(OH)_2_ was close.

The TGA and DTG curves of the control group and E_9_ group at 3 d are shown in Figure 13b; the peak of C-S-H gel and AFt was more obvious in the E_9_ group, and the volume deformation of the E_9_ group was expansion, whereas that of the control group was contraction. On the one hand, the substitution of some cement by EA reduces the cement clinker C3S involved in the reaction, and the cement hydration reaction is smaller; on the other hand, because EA participates in the hydration reaction and competes with the cement hydration reaction for moisture, generating more C-S-H gel and AFt, so the macro volume deformation of E_9_ group is expansion.

TGA and DTG curves of the Ref. and E_9_ groups at 7 d are shown in Figure 13c; group E_9_ had more C-S-H gels and AFt. Compared with that at 3 d, the peak of CH in the 7 d E_9_ group was weakened, while the peak of carbonate was increased, indicating that some Ca(OH)_2_ reacts with CO_2_ in the air to form CaCO_3_. Moreover, the E_9_ group had more carbonate content compared with the Ref. group, and this indicated that the E_9_ group produced more Ca(OH)_2_, so it was easier to react with CO_2_ to produce CaCO_3_, which can also explain the lower compressive strength of the E_9_ group than the Ref. group at the same age. The TGA curve of the E_9_ group gradually approached that of the Ref. group with the increase in age, the reason was that the gradual formation of more C-S-H gels and AFt in the Ref. group, while the E_9_ group reacted with aluminate ion and changed to AFm [49].

**Figure 14:**
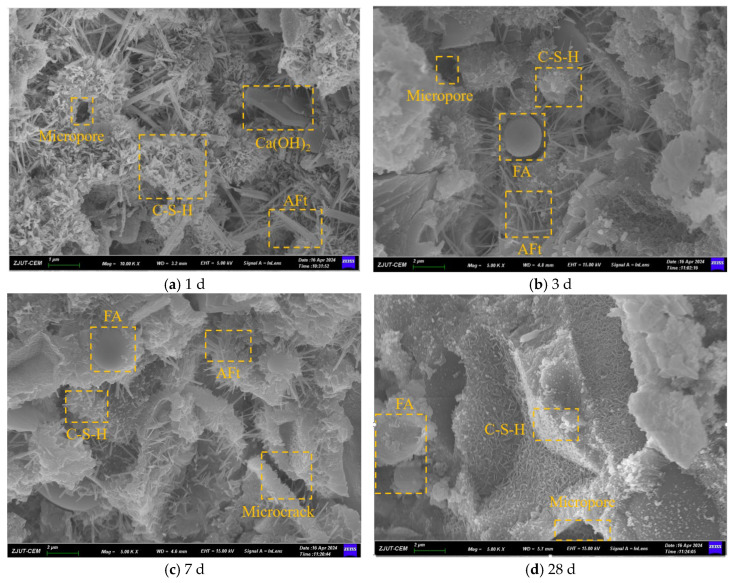
SEM images of Ref. groups at different ages.

The paste micromorphology of the Ref. group at different ages is shown in Figure 14. It can be seen from Figure 14 that the un-hydrated cement material also existed in the SEM images of the control samples at the age of 1 d, the reason was the relatively low-water glue, and there were more un-hydrated cement particles in the paste. A small amount of needle rod calcium alum stone, and fibrous C-S-H gel were generated in the paste of the control group under closed conditions. The structure at 3 days had more fine pores and cracks, which may be due to the formation of calcium hydroxide and external hydration products through the dissolution and deposition of cement clinker in the early hydration. For example, fine needles can be seen around the fly ash particles and in the holes, which are less limited by space, so the structure is loose. The increase in C-S-H gel density at 7 d shows further polymerization between C-S-H gels and increased hydration degree [51]. The C-S-H gel on the surface of fly ash particles also began to increase, and the surface became rough, indicating that fly ash began to erode, but its hydration degree is not high. At this time, small cracks and pores can be seen, indicating that the concrete is in a state of shrinkage due to hydration. At 28 d, the amount of it was decreased, probably due to partial dissolution or transformation. The C-S-H gels on the FA surface continued to increase, while the C-S-H gels changed from fibrous to large mass structures, indicating that the progress of the hydration reaction continued. The formation of the mass C-S-H gel means that the cement matrix becomes denser and more stable, and the number of needle-shaped AFt in the sample decreases significantly, due to the conversion of AFt to monosulfur hydrated calcium aluminate (AFm) at high calcium hydroxide concentration, which significantly enhances the compressive strength of the concrete [52,53,54].

### 4.3. Effect of Combined Use of EA and SRA

**Figure 15:**
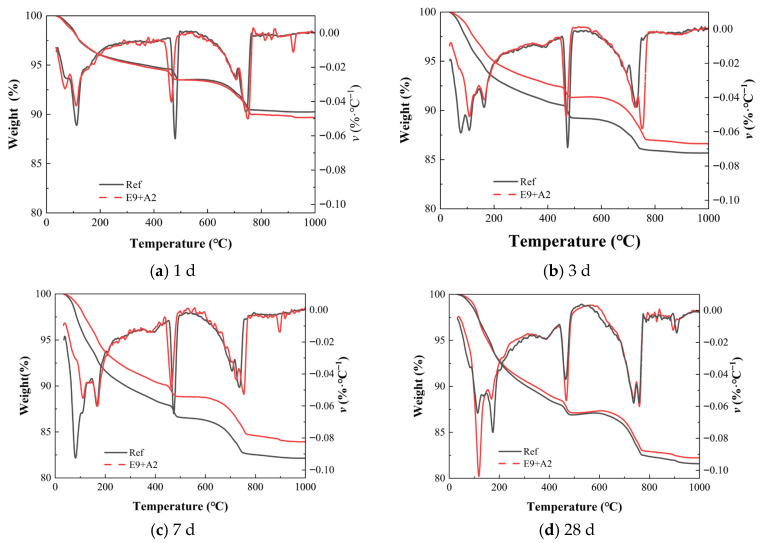
Thermogravimetry analysis of paste containing EA and SRA at different ages.

To analyze the effect of remixed EA and SRA on hydration products in concrete, a series of TG analysis tests was performed on Ref. and E_9+A2_ groups at 1 d, 3 d, 7 d, and 28 d, and the experimental results are shown in Figure 15.

The TGA and DTG curves of the 1 d age period of the Ref. and E_9+A2_ groups were shown in Figure 15a, it can be seen that the TGA curves of the Ref. and E_9+A2_ groups were similar, and the total mass loss trend of the E_9+A2_ group was slightly higher than that of the Ref. group, which indicates that the incorporation of SRA delayed the hydration reaction of EA, and less C-S-H gels and Aft were generated. Although the incorporation of SRA did not completely inhibit cement hydration, it did significantly affect the hydration reaction of EA, resulting in TGA curves showing fewer hydration products.

The TGA and DTG curves at 3 d and 7 d of the Ref. and E_9+A2_ groups are shown in Figure 15b,c. The results showed that the overall trend of the curves between E_9+A2_ and Ref. groups was similar, but different in weight loss and reaction rate. Two loss peaks between room temperature and 500 °C showed that the Ref. group produced more C-S-H gel and Ca(OH)_2_ at the beginning of hydration. However, the E_9+A2_ group lost less weight in the same temperature range, indicating that its hydration reaction was slower and it generated fewer hydration products. At 7 d, the curve difference between the E_9+A2_ group and the Ref. group remained, but the gap was relatively reduced. The TGA curves in Figure 15 showed a similar weight loss process in the E_9+A2_ group and the Ref. group, but the total weight loss was still lower than that in the Ref. group. This indicates that although the cement hydration proceeds to generate a certain amount of hydration product, the rate of cement hydration was changed due to the incorporation of SRA. The main component of SRA was polyether organic compounds, whose molecules would adsorb on the surface of cement particles and form a thin film. This adsorption would temporarily hinder the contact between cement particles and water, and delay the early hydration reaction rate, leading to the slower formation rate of C-S-H gel and other hydration products [51]. Because the early strength mainly depends on the rapid accumulation of hydration products, the reduction in hydration rate directly affects the compressive strength of early concrete. These results could further explain the difference in compressive strength (the E_9+A2_ group was 37.5% and 29.7%, and lower than the E_9_ group). The cement hydration of the E_9+A2_ group was relatively slow, resulting in fewer hydration products generated in the early stage, especially in the formation of C-S-H gel. The number and distribution of C-S-H gels had a direct effect on the compressive strength of the concrete, so that the E_9+A2_ group was lower at 3 d and 7 d.

**Figure 16:**
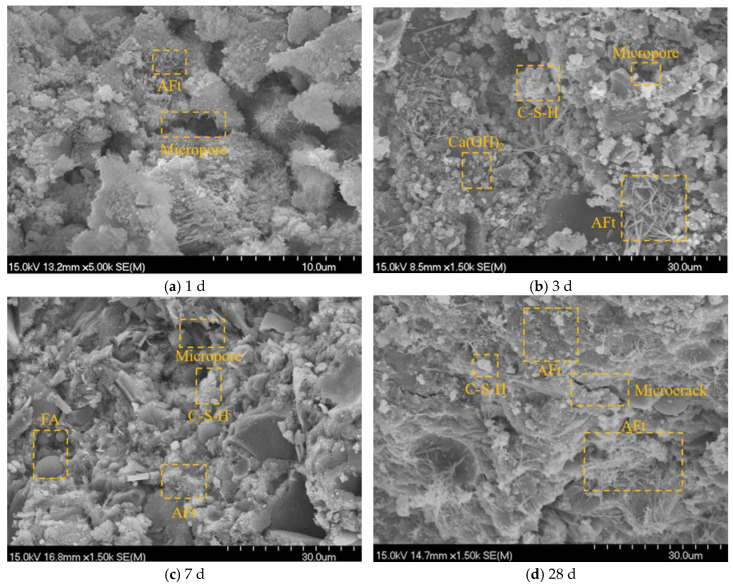
SEM images of E_9+A2_ groups at different ages.

The morphology of the E_9+A2_ group was shown in Figure 16. The needle Aft crystals of the E_9+A2_ group at 1 d and 3 d were smaller compared with the Ref. group, because the incorporation of SRA slowed the cement hydration, and the microcracks in the transition zone were smaller, and more Aft and Ca(OH)_2_ crystals were distributed in the micro cracks and micro pores, and the volume shrinkage was reduced. In the microstructure image at 28 d of the E_9_ group, the smaller the size of the hydration product, and the tighter the microstructure of the hardened paste, the compressive strength at 28 d was also improved. At the same time, the number of hydration products was more and more dense, which may be due to the incorporation of SRA, which makes the hydration reaction of gelentiate material more sufficient. When reducing the contraction caused by hydration, more Aft, Ca(OH)_2_ crystals are generated to fill microcracks and pores, so the volume deformation and expansion strain also increase [55,56].

### 4.4. Analysis of the Mechanism of Volume Deformation Regulation

A PSO-BPNN model was developed based on autogenous shrinkage data of concrete with various mix proportions collected from literature, aiming to predict the autogenous shrinkage behavior of unregulated concrete. The prediction accuracy of the model was validated using experimental data. Results show that the PSO-BPNN model exhibits higher accuracy, with a prediction error of less than 10% at 28 days, demonstrating strong generalization capability.

The effects of different dosages of expansive agent and shrinkage-reducing admixture on the workability, setting time, compressive strength, and volume deformation of concrete were investigated through experiments. The results indicate that the incorporation of EA significantly improves the volume stability of concrete, achieving an expansive compensation of 99.9 × 10^−6^ at a dosage of 9%. However, excessive EA dosage reduces both workability and compressive strength. SRA improves concrete flowability and enhances compressive strength at later ages, though it may suppress early strength development.

The development of volume deformation in concrete under both unrestrained and steel tube-restrained conditions was compared. Experimental results showed that the lateral confinement provided by the steel tube effectively suppresses the development of core concrete shrinkage, resulting in a significantly reduced final shrinkage value. Analysis of internal stress evolution in SCC indicates that volume deformation mainly affects stress distribution during the early expansion phase.

The influence of EA and SRA on the microstructure of concrete was analyzed using scanning electron microscopy and thermogravimetric analysis. Results reveal that EA promotes the early formation of AFt and Ca(OH)_2_, enhancing the expansion capacity of hydration products and improving shrinkage compensation [44]. In contrast, SRA delays hydration reactions and suppresses early formation of C-S-H gel, but allows for more complete hydration at later stages, leading to recovery of compressive strength at 28 days.

## 5. Conclusions

In this study, a PSO-BPNN model was constructed, and the concrete autogenous shrinkage strain before regulation was predicted based on PSO-BPNN. Compensating for shrinkage strain based on prediction by expansion or by a combination of expansion and contraction was investigated. The major findings are as follows:

1. The PSO-BPNN model has a good consistency between predicted and measured values, demonstrating PSO-BPNN the model’s high accuracy in predicting concrete autogenous shrinkage. The prediction error of the PSO-BPNN model was less than 10% at 28 d, demonstrating strong generalization capability. The prediction was a guide for regulation to compensate for shrinkage, and the experimental workload was reduced. But the level of PSO-BPNN model intelligence remains relatively low. With the increase in relevant engineering and experimental data, model accuracy will be further improved. Meanwhile, predictive algorithms such as PSO-BPNN would be further optimized with the advancement of AI technology.
2. The reasonable regulation of the amount of expansion agent and reducing shrinkage agent is the key to ensuring the stability of concrete volume, especially in structures that need to control the autogenous shrinkage.
3. The composite incorporation with EA and SRA can significantly change the volume deformation of steel tube constrained concrete, and the amount of SRA has a significant impact on the volume stability of concrete. While the EA plays the role of compensating for autogenous shrinkage, 2% of SRA can also reduce the shrinkage deformation of concrete, and the synergy between EA and SRA in concrete volume shrinkage deformation was good. Reasonable control of the mixing amount of SRA can effectively balance the autogenous shrinkage and expansion effect of concrete and improve the volume stability and long-term durability of concrete.

## Figures and Tables

**Table 1:** Mix proportion of control concrete (kg/m^3^).

№	W/B	C	FA	M	S	G	W	SP
Ref	0.32	290	97	97	722	1039	155	5.8

Note: W/B, C, FA, M, S, G, W, and SP in the table, respectively, represent water-to-cement ratio, cement, fly ash, ground granulated blast furnace slag (GGBFS), sand, coarse aggregate, water, and high-range water reducer (superplasticizer).

**Table 2:** Mix proportion of regulated concrete (kg/m^3^).

№	C	FA	M	S	G	W	SP	EA	SRA
Ref	290	97	97	722	1039	155	5.8	—	—
E8	251	97	97	722	1039	155	5.8	38.7	—
E9	247	97	97	722	1039	155	5.8	43.5	—
E10	242	97	97	722	1039	155	5.8	48.4	—
A1	290	97	97	722	1039	155	5.8	—	4.84
A2	290	97	97	722	1039	155	5.8	—	9.68
E9A1	247	97	97	722	1039	155	5.8	43.5	4.84
E9A2	247	97	97	722	1039	155	5.8	43.5	9.68

## Data Availability

The data that support the findings of this study are available from the https://www.cnki.net (1 April 2026) and https://webofscience.clarivate.cn/wos (1 April 2026).

## Supplementary Materials

The following supporting information can be downloaded at: https://www.mdpi.com/article/10.3390/ma19071468/s1, Table S1: Database.
